# Supplementary material for: Multi-Omics Approaches for Liver Reveal the Thromboprophylaxis Mechanism of Aspirin Eugenol Ester in Rat Thrombosis Model
Source: Int J Mol Sci. 2024 Feb 10;25(4):2141. doi: 10.3390/ijms25042141 (PMC10889733; doi:10.3390/ijms25042141)
Supplement: Supplementary file 1 [file ijms-25-02141-s001.zip › Supplementary Materials Figures and Tables.docx]

**Figure S1.** Basic information statistics of rat liver proteomics by iTRAQ analysis. PSM: peptide spectrum match. Total spectra are the secondary mass spectra, and spectra (PSM) are the secondary mass spectra after quality control. Unique Peptides are the identified peptides that belong only to a group of proteins.

**Figure S2.** Venn diagram showing the overlap of different abundant proteins.

**Figure S3.** GO enrichment analysis of the DEPs. (**A**) Control group *vs* model group. (**B**) ASA group *vs* model group. (**C**) EUG group *vs* model group. (**D**) AEE group *vs* model group.

**Table S1.** Summary of the sequencing reads from the liver samples in different groups.

| Sample ID | Raw reads | Clean reads | Average length | Clean reads % | Q20 % | Q30 % | Total mapped | Mapped ratio (%) |
| --- | --- | --- | --- | --- | --- | --- | --- | --- |
| AEE1 | 76684660 | 74993196 | 144.0722 | 97.79% | 96.95% | 91.86% | 69476326 | 92.60% |
| AEE2 | 79791600 | 77914364 | 143.9394 | 97.65% | 96.95% | 91.81% | 71757068 | 92.10% |
| AEE3 | 90489288 | 88256070 | 143.8471 | 97.53% | 96.81% | 91.37% | 81804048 | 92.70% |
| ASA1 | 72983760 | 71098108 | 143.7445 | 97.42% | 96.71% | 91.27% | 66231908 | 93.20% |
| ASA2 | 80161858 | 77984254 | 143.5706 | 97.28% | 96.76% | 91.32% | 72112634 | 92.50% |
| ASA3 | 89463582 | 87145092 | 143.5273 | 97.41% | 96.75% | 91.46% | 80790586 | 92.70% |
| Model1 | 84138774 | 81466074 | 143.2503 | 96.82% | 96.56% | 90.98% | 75582674 | 92.80% |
| Model2 | 78780466 | 76860098 | 143.9066 | 97.56% | 96.80% | 91.51% | 70670340 | 91.90% |
| Model3 | 96759692 | 94231930 | 144.1401 | 97.39% | 96.71% | 91.32% | 87089116 | 92.40% |
| Eugenol1 | 71816170 | 69937424 | 143.9435 | 97.38% | 96.71% | 91.38% | 64838843 | 92.70% |
| Eugenol2 | 1.17E+08 | 1.14E+08 | 143.1188 | 97.56% | 96.80% | 91.45% | 105358692 | 92.30% |
| Eugenol3 | 80962006 | 78804102 | 143.4782 | 97.33% | 96.66% | 91.22% | 72538533 | 92% |
| Control_1 | 86486336 | 84063062 | 143.9298 | 97.20% | 96.71% | 91.32% | 77693865 | 92.40% |
| Control_2 | 71723930 | 70100572 | 144.2641 | 97.74% | 97.05% | 92.06% | 64413399 | 91.90% |
| Control_3 | 80249790 | 78231858 | 144.0091 | 97.49% | 96.81% | 91.52% | 72549649 | 92.70% |

**Table S2.** Summary statistics of different abundant proteins.

| Comparisons | Up-regulated | Down-regulated | All |
| --- | --- | --- | --- |
| Model/Control | 240 | 358 | 598 |
| ASA/Model | 159 | 83 | 242 |
| Eug/Model | 39 | 91 | 130 |
| AEE/Model | 50 | 109 | 159 |

Ratios with *P*-values < 0.05 and fold changes of > 1.2 or < 0.83 were considered as different abundant proteins.

**Table S3.** Pathway analysis results of the metabolites in the liver tissue with MetaboAnalyst.

|  | Total | Expected | Hits | Raw P | -Log (P) | Holm adjust | FDR | Impact |
| --- | --- | --- | --- | --- | --- | --- | --- | --- |
| Aminoacyl-tRNA biosynthesis | 48 | 1.2406 | 10 | 1.29E-07 | 15.866 | 1.08E-05 | 1.08E-05 | 0.3448 |
| Histidine metabolism | 16 | 0.41352 | 4 | 0.000555 | 7.4957 | 0.046104 | 0.022907 | 0.33334 |
| Valine, leucine and isoleucine biosynthesis | 8 | 0.20676 | 3 | 0.000818 | 7.1085 | 0.067084 | 0.022907 | 0.25 |
| beta-Alanine metabolism | 21 | 0.54274 | 4 | 0.001664 | 6.3988 | 0.13475 | 0.031225 | 0.04762 |
| Biosynthesis of unsaturated fatty acids | 36 | 0.93042 | 5 | 0.001859 | 6.2879 | 0.14869 | 0.031225 | 0 |
| Phenylalanine, tyrosine and tryptophan biosynthesis | 4 | 0.10338 | 2 | 0.003781 | 5.5778 | 0.29868 | 0.052435 | 0.75 |
| Arginine biosynthesis | 14 | 0.36183 | 3 | 0.004774 | 5.3447 | 0.37233 | 0.052435 | 0.25 |
| Glutathione metabolism | 28 | 0.72366 | 4 | 0.004994 | 5.2996 | 0.38452 | 0.052435 | 0.13514 |
| Pantothenate and CoA biosynthesis | 19 | 0.49105 | 3 | 0.011619 | 4.4552 | 0.88301 | 0.10844 | 0.11112 |
| Arginine and proline metabolism | 38 | 0.98211 | 4 | 0.014946 | 4.2033 | 1 | 0.12554 | 0.15 |
| Alanine, aspartate and glutamate metabolism | 28 | 0.72366 | 3 | 0.033462 | 3.3974 | 1 | 0.25542 | 0.32258 |
| Phenylalanine metabolism | 12 | 0.31014 | 2 | 0.036488 | 3.3108 | 1 | 0.25542 | 0.6 |
| Butanoate metabolism | 15 | 0.38767 | 2 | 0.055295 | 2.8951 | 1 | 0.33177 | 0.06667 |
| Nicotinate and nicotinamide metabolism | 15 | 0.38767 | 2 | 0.055295 | 2.8951 | 1 | 0.33177 | 0.09524 |
| Valine, leucine and isoleucine degradation | 40 | 1.0338 | 3 | 0.081662 | 2.5052 | 1 | 0.4573 | 0.05661 |
| Citrate cycle (TCA cycle) | 20 | 0.5169 | 2 | 0.092325 | 2.3824 | 1 | 0.4847 | 0.13794 |
| Sphingolipid metabolism | 21 | 0.54274 | 2 | 0.10042 | 2.2984 | 1 | 0.49621 | 0.15625 |
| Linoleic acid metabolism | 5 | 0.12922 | 1 | 0.12287 | 2.0966 | 1 | 0.57339 | 0.75 |
| Lysine degradation | 25 | 0.64612 | 2 | 0.13461 | 2.0054 | 1 | 0.58246 | 0.1 |
| D-Glutamine and D-glutamate metabolism | 6 | 0.15507 | 1 | 0.14561 | 1.9268 | 1 | 0.58246 | 0.25 |
| Nitrogen metabolism | 6 | 0.15507 | 1 | 0.14561 | 1.9268 | 1 | 0.58246 | 0.25 |
| Glyoxylate and dicarboxylate metabolism | 32 | 0.82704 | 2 | 0.19931 | 1.6129 | 1 | 0.761 | 0.07692 |
| Biotin metabolism | 10 | 0.25845 | 1 | 0.23098 | 1.4654 | 1 | 0.84359 | 0 |
| Pyrimidine metabolism | 39 | 1.008 | 2 | 0.26708 | 1.3202 | 1 | 0.93477 | 0.01695 |
| alpha-Linolenic acid metabolism | 13 | 0.33598 | 1 | 0.2895 | 1.2396 | 1 | 0.97271 | 0.125 |
| Ether lipid metabolism | 20 | 0.5169 | 1 | 0.40967 | 0.89241 | 1 | 1 | 0.10526 |
| Pyruvate metabolism | 22 | 0.56859 | 1 | 0.44019 | 0.82054 | 1 | 1 | 0.11111 |
| Propanoate metabolism | 23 | 0.59443 | 1 | 0.45488 | 0.78773 | 1 | 1 | 0.03846 |
| Purine metabolism | 66 | 1.7058 | 2 | 0.51619 | 0.66128 | 1 | 1 | 0.03409 |
| Porphyrin and chlorophyll metabolism | 30 | 0.77535 | 1 | 0.54765 | 0.60212 | 1 | 1 | 0.03846 |
| Cysteine and methionine metabolism | 33 | 0.85288 | 1 | 0.58252 | 0.54039 | 1 | 1 | 0.0303 |
| Glycerophospholipid metabolism | 36 | 0.93042 | 1 | 0.61477 | 0.48651 | 1 | 1 | 0.03846 |
| Arachidonic acid metabolism | 36 | 0.93042 | 1 | 0.61477 | 0.48651 | 1 | 1 | 0.36111 |
| Fatty acid elongation | 39 | 1.008 | 1 | 0.64458 | 0.43915 | 1 | 1 | 0 |
| Fatty acid degradation | 39 | 1.008 | 1 | 0.64458 | 0.43915 | 1 | 1 | 0.02041 |
| Primary bile acid biosynthesis | 46 | 1.1889 | 1 | 0.70568 | 0.3486 | 1 | 1 | 0.02128 |
| Fatty acid biosynthesis | 47 | 1.2147 | 1 | 0.71352 | 0.33754 | 1 | 1 | 0.01471 |

Total: The total number of compounds in the pathways; the hits are the actually matched number from the user upload data; the raw*P* is the original *P* value calculated from the enrichment analysis; the impact is the pathway impact value calculated from pathway analysis. The metabolome view on the left shows all matched pathways according to the *P* values from the pathway enrichment analysis and pathway impact values from the pathway topology analysis.
